# Development of health-based exposure limits for radiofrequency radiation from wireless devices using a benchmark dose approach

**DOI:** 10.1186/s12940-021-00768-1

**Published:** 2021-07-17

**Authors:** Uloma Igara Uche, Olga V. Naidenko

**Affiliations:** Environmental Working Group, 1250 I Street NW, Suite 1000, Washington, DC, 20005 USA

**Keywords:** Radiofrequency radiation, Specific Absorption Rate, SAR, Exposure guidelines, Benchmark modeling

## Abstract

**Background:**

Epidemiological studies and research on laboratory animals link radiofrequency radiation (RFR) with impacts on the heart, brain, and other organs. Data from the large-scale animal studies conducted by the U.S. National Toxicology Program (NTP) and the Ramazzini Institute support the need for updated health-based guidelines for general population RFR exposure.

**Objectives:**

The development of RFR exposure limits expressed in whole-body Specific Absorption Rate (SAR), a metric of RFR energy absorbed by biological tissues.

**Methods:**

Using frequentist and Bayesian averaging modeling of non-neoplastic lesion incidence data from the NTP study, we calculated the benchmark doses (BMD) that elicited a 10% response above background (BMD_10_) and the lower confidence limits on the BMD at 10% extra risk (BMDL_10_). Incidence data for individual neoplasms and combined tumor incidence were modeled for 5% and 10% response above background.

**Results:**

Cardiomyopathy and increased risk of neoplasms in male rats were the most sensitive health outcomes following RFR exposures at 900 MHz frequency with Code Division Multiple Access (CDMA) and Global System for Mobile Communications (GSM) modulations. BMDL_10_ for all sites cardiomyopathy in male rats following 19 weeks of exposure, calculated with Bayesian model averaging, corresponded to 0.27–0.42 W/kg whole-body SAR for CDMA and 0.20–0.29 W/kg for GSM modulation. BMDL_10_ for right ventricle cardiomyopathy in female rats following 2 years of exposure corresponded to 2.7–5.16 W/kg whole-body SAR for CDMA and 1.91–2.18 W/kg for GSM modulation. For multi-site tumor modeling using the multistage cancer model with a 5% extra risk, BMDL_5_ in male rats corresponded to 0.31 W/kg for CDMA and 0.21 W/kg for GSM modulation.

**Conclusion:**

BMDL_10_ range of 0.2—0.4 W/kg for all sites cardiomyopathy in male rats was selected as a point of departure. Applying two ten-fold safety factors for interspecies and intraspecies variability, we derived a whole-body SAR limit of 2 to 4 mW/kg, an exposure level that is 20–40-fold lower than the legally permissible level of 0.08 W/kg for whole-body SAR under the current U.S. regulations. Use of an additional ten-fold children’s health safety factor points to a whole-body SAR limit of 0.2–0.4 mW/kg for young children.

**Supplementary Information:**

The online version contains supplementary material available at 10.1186/s12940-021-00768-1.

## Introduction

The health risk assessment of non-ionizing electromagnetic radiation generated science and policy debates for decades, particularly around the health effects of radiofrequency radiation (RFR) in the 3 kHz to 300 GHz frequency range used for wireless communications [1–4]. Among the reported biological effects of electromagnetic fields are harm to fetal growth and development [5–13], changes in heart rate variability [14, 15], changes in brain activity [16, 17], and elevated risk of several cancers [18–21]. In 2011, the International Agency for Research on Cancer (IARC) classified radiofrequency electromagnetic fields as “possibly carcinogenic to humans” based on an increased risk of glioma, a malignant brain cancer, associated with cellular phone use [18].

The mechanisms by which radiofrequency radiation affects cells, tissues, and organisms are not well understood and may include diverse processes such as inhibition of the mitotic spindle apparatus leading to impaired cell division and cell death [22], changes in the activity of voltage-gated calcium channels [23–26], changes in the concentrations of reactive oxygen species and redox homeostasis [25, 27–32], changes in intracellular enzymes and gene expression [33], and changes in membrane permeability [34]. DNA damage following exposure to RFR [35, 36] may be a direct effect or due to secondary mechanisms, such as interference with DNA repair processes [37]. As summarized in a recent review, these biological effects of RFR occur without substantial temperature increases in tissues [27]. Highlighting the complexity of these biological interactions, some forms of electromagnetic fields, described as “tumor treating fields” in the medical research literature, are now explored as a potential therapy for glioblastoma and other cancers [22, 38].

With a continuously growing variety of RFR sources in the everyday environment and drastically increased intensity of daily exposure to RFR from personal wireless devices and from short- and long-range sources such as Wi-Fi routers, cell phone towers, and small cell transmitters, the question of safe exposure levels for the public, especially children, is urgent and important. In the U.S., general population exposure standards for RFR were set by the Federal Communications Commission in 1996 based on studies conducted in the 1970s and 1980s in which behavior changes were observed in laboratory animals exposed to RFR for the duration of minutes to hours [2, 39, 40]. For the frequency range of 100 kHz to 6 GHz, the U.S. standards define the legally allowable exposures in terms of Specific Absorption Rate, or SAR, which refers to the relative amount of energy absorbed by biological tissues. For non-occupational exposures, U.S. regulations allow a SAR of 0.08 W/kg averaged over the whole body and a localized peak spatial SAR of 1.6 W/kg averaged over 1 g of tissue. Localized peak SAR limits apply to wireless device use in immediate contact with the body, for example a cellular phone held near the head.

In the past two decades, epidemiological studies reported an elevated risk of gliomas, acoustic neuromas, parotid gland tumors, and thyroid cancers in regular cellular phone users. The challenge of ascertaining the length, frequency, and intensity of wireless device use and continuously changing communication technologies make it difficult to develop a precise exposure metric from epidemiological research. Recent studies of RFR in laboratory animals provide much-needed information on the specific RFR exposure levels associated with elevated risks of adverse health effects [41]. The results from two long-term, large-scale studies of RFR in laboratory rodents, the study from the U.S. National Toxicology Program (NTP) [42] and the study from the Ramazzini Institute in Italy [7, 43], are especially informative. The NTP study examined the health effects of the RFR exposure of interim duration (19 weeks, including the prenatal period) and long-term exposure (2 years) and reported cardiotoxic, genotoxic, and carcinogenic effects [35, 44–46]. In the Ramazzini Institute study, rats were exposed from prenatal life until natural death, and a statistically significant increase in the incidence of heart schwannomas was reported [7]. Here we model the health effects incidence data from the National Toxicology Program (NTP) to estimate the departure points for exposure guidelines and to open the discussion of exposure limits for wireless devices that would protect the health of vulnerable populations, especially children.

## Methods

Lesion incidence data were accessed from the National Toxicology Program (NTP) reports [20, 21]. The NTP examined the effects of RFR with two different modulations, Code Division Multiple Access (CDMA) and Global System for Mobile Communications (GSM), and in two species: rats and mice, exposed to 900 MHz and 1900 MHz radiation, respectively [44, 45]. Animals were exposed every day for the duration of the analysis, and the length of daily RFR exposure totaled 9 h, 10 min per day, achieved via 18 h, 20 min daily exposure in 10-min on–off cycles. The study design for the two species differed with respect to early life exposure. Rats were exposed from the prenatal period, starting on gestation day 5, during the 3-week lactation period, and continuing for the rest of the two-year study duration, with whole-body SAR levels of 1.5, 3, and 6 W/kg [44]. For mice, daily exposure with whole-body SAR levels of 2.5, 5, or 10 W/kg started at 5–6 weeks of age, around the age of puberty in this species [45]. The NTP study reported an increased incidence of cardiomyopathy in female and male rats and increased incidences of various neoplasms in male rats [44]. In contrast, similar effects were not observed in mice [45]. For the analysis here, we focused on the data from rats because that dataset reflects the potential health impacts of life-long exposure to radiofrequency radiation starting from the prenatal period.

Benchmark dose modeling for non-neoplastic and neoplastic incidence data was conducted using U.S. EPA Benchmark Dose Software (BMDS) version 3.2 (https://www.epa.gov/bmds). For non-neoplastic effects, we used both frequentist and Bayesian model averaging options within the BMDS software. For neoplastic effects, we used the frequentist multistage cancer model (MS Combo) following the U.S. EPA’s technical guidance for modeling tumor data [47]. The benchmark approach considers the response at all doses tested in the study and can help fill the data gap regarding the shape of the dose–response curve below the lowest dose tested. As discussed in the benchmark dose modeling research literature [48–50], the best fitting models were selected based on the lowest Akaike Information Criterion, goodness-of-fit p values, scaled residual, and visual inspection of the curve fit.

For benchmark modeling, the choice of the appropriate percentage change in the response above the background depends on the specific dataset [47]. Peer-reviewed literature and guidance documents on BMD modeling published by authoritative agencies have used BMD modeling with 5% or 10% extra risk [47, 48, 50]. Here we applied the standard approach of calculating the benchmark dose (BMD_10_) at a 10% benchmark response for non-neoplastic incidence data. For neoplastic incidence data, we conducted modeling of both 5% and 10% benchmark response above the background and selected the 5% response rate (BMD_5_) as an approach that allowed for successful modeling of the neoplasm incidence data from the NTP study. BMDS software also calculates the 95% lower confidence limit (BMDL) on the exposure level that would produce a response at a selected incidence frequency above the background [47]. BMDL values can be used as a point of departure for the development of health-based guidelines and risk assessment.

## Results

Analysis of dose–response patterns for different lesions observed in the NTP study suggests that some health effects associated with RFR exposures exhibit non-monotonic dose–response relationships that may be influenced by gender and RFR modulation. RFR exposure corresponding to whole-body SAR of 1.5 W/kg, the lowest tested level in the NTP study, was associated with an increase in the incidence of various nonneoplastic and neoplastic effects relative to sham-exposed animals, indicating that “No Observed Adverse Effect Level”, or NOAEL, was not identified in the NTP study. For some health outcomes, the highest incidence in the RFR-exposed versus control group was observed at 1.5 W/kg exposure, with a decrease in incidence at SAR values of 3 and 6 W/kg.

**Fig. 1:**
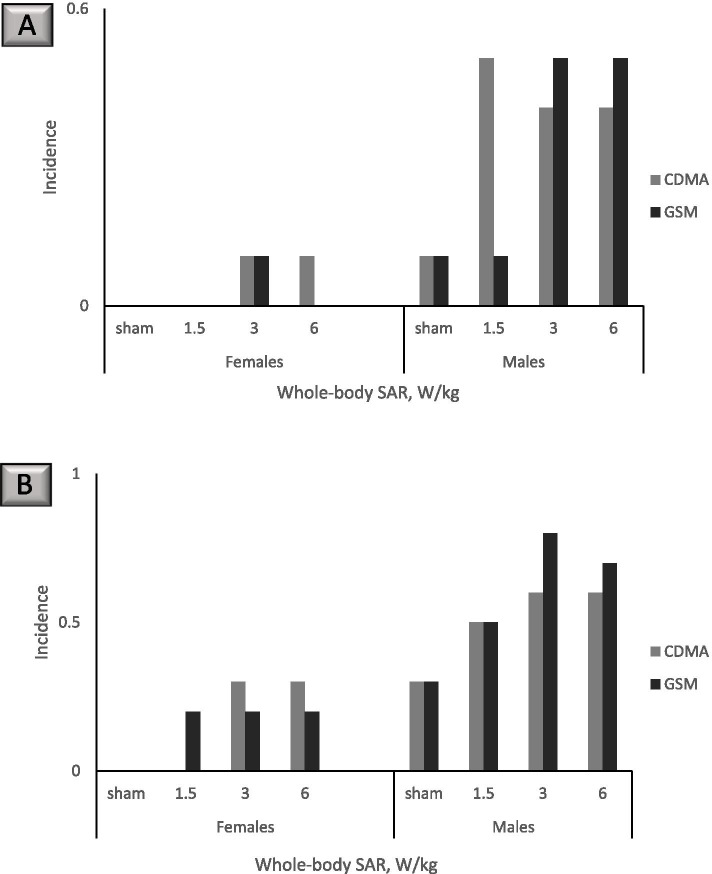
Cardiomyopathy incidence at 19 weeks. Each experimental group had 10 animals. Where no bar is shown, the specific outcome in question was not observed in the animal group. **A** Right ventricle cardiomyopathy. **B** All sites cardiomyopathy

**Fig. 2:**
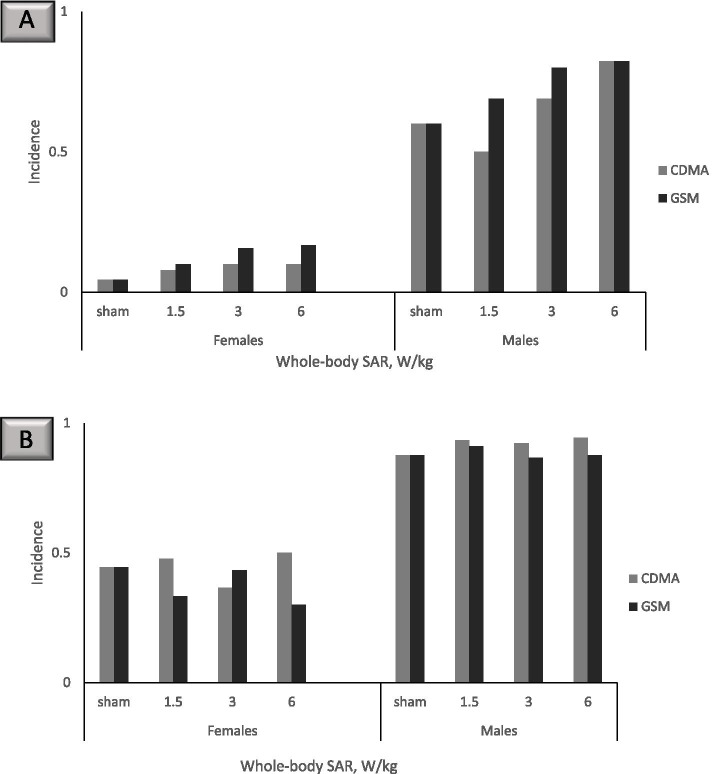
Cardiomyopathy incidence at 2 years. Each experimental group had 90 animals. **A** Right ventricle cardiomyopathy. **B** All sites cardiomyopathy

The findings for cardiomyopathy are particularly compelling. Cardiomyopathy is defined in the NTP report as the “*degeneration and necrosis of myofibers with a mild inflammatory response of macrophages and lymphocytes with occasional neutrophils* [44]*”*. While observed in both males and females and at both RFR modulations, dose–response relationships were distinct for right ventricle versus all sites cardiomyopathy. At 19 weeks of exposure, CDMA-exposed male rats had a lower incidence of right ventricle cardiomyopathy in the 3 and 6 W/kg dose groups compared to the 1.5 W/kg dose group, an effect that was not observed in GSM-exposed male rats at the same time point (Fig. 1A). For all sites cardiomyopathy, male rats in the highest-dose GSM group (SAR 6 W/kg) had lower incidence compared to male rats in the second-highest dose group of 3 W/kg, while for CDMA-exposed male rats all sites cardiomyopathy incidence was the same for the 3 and 6 W/kg groups (Fig. 1B). At 19 weeks, cardiomyopathy incidence in females was lower than in males and did not exhibit a dose–response relationship (Fig. 1). At 2 years, right ventricle cardiomyopathy in both CDMA- and GSM exposed rats exhibited a visible dose–response relationship (Fig. 2A), which was not observed for all sites cardiomyopathy (Fig. 2B). Overall, increased incidence of cardiomyopathy both at the interim examination point of 19 weeks and at the end of the study reinforces the health significance of the findings.

**Fig. 3:**
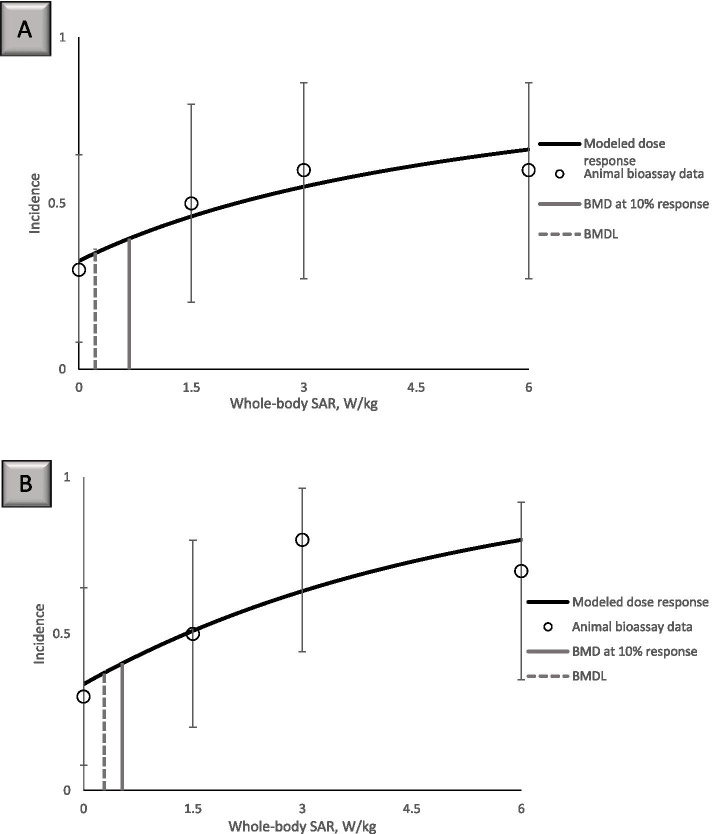
Benchmark dose modeling of all sites cardiomyopathy in male rats at 19 weeks. **A** Fitting of the data for CDMA exposures to the log-logistic model results in BMD_10_ of 0.67 W/kg and BMDL_10_ of 0.22 W/kg. **B** Fitting of the data for GSM exposures to the Weibull model results in BMD_10_ of 0.54 W/kg and BMDL_10_ of 0.24 W/kg

**Fig. 4:**
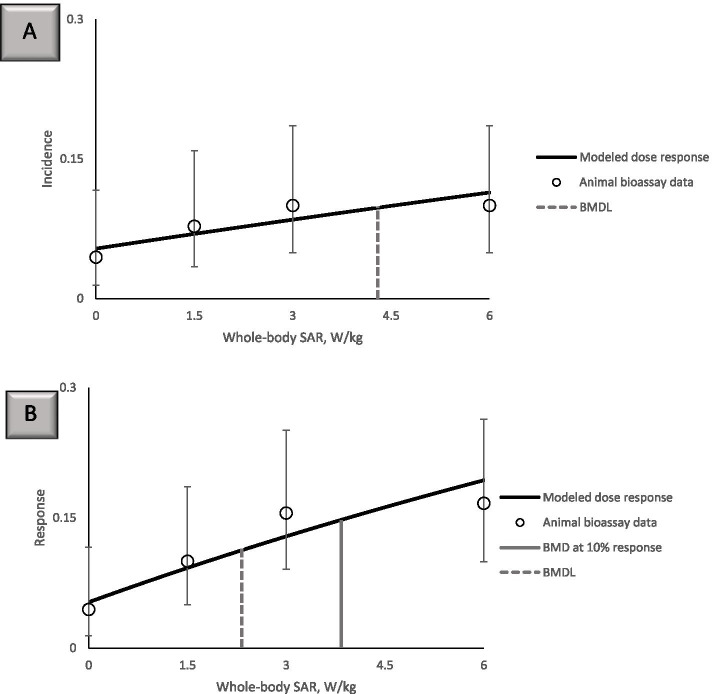
Benchmark dose modeling of right ventricle cardiomyopathy in female rats at 2 years. **A** Fitting of the data for CDMA exposures to the log-logistic model results in BMD_10_ of 9.80 W/kg (above the highest dose of 6 W/kg tested in the NTP study, and thus not shown in the panel) and BMDL_10_ of 4.30 W/kg. **B** Fitting of the data for GSM exposures to the log-logistic model results in BMD_10_ of 3.83 W/kg and BMDL_10_ of 2.32 W/kg

We conducted BMDS modeling for all available cardiomyopathy datasets from the NTP study. Successful model fitting was observed for all sites cardiomyopathy in male rats at 19 weeks of exposure and for right ventricle cardiomyopathy in both male and female rats at the 2-year point (Supplementary Table 1). Figures 3 and 4 illustrate the modeling results for all sites cardiomyopathy in male rats at 19 weeks for CDMA (Fig. 3A) and GSM modulations (Fig. 3B) and right ventricle cardiomyopathy in female rats at 2 years for CDMA (Fig. 4A) and GSM (Fig. 4B). Different BMDS frequentist models resulted in similar or identical BMD values and comparable quality of data fit (Supplementary Table 1). Due to the lack of biological information for determining which mathematical model would be most appropriate for the datasets analyzed here, we used the Bayesian model averaging approach to define the BMD_10_ and BMDL_10_ values. Since the highest variability in the dose–response relationship was observed at the highest SAR exposure dose, we conducted modeling with and without this data point (Supplementary Table 2 and Table 1).

While BMD modeling of the datasets without the highest dose of 6 W/kg SAR generally resulted in better model fit, overall BMD and BMDL values were comparable for modeling with and without the highest dose (Supplementary Table 1). For male rats at 19 weeks of exposure, the most sensitive responses were observed for GSM, with 0.2–0.29 w/kg BMDL_10_, compared to 0.27–0.42 W/kg BMDL_10_ value for CDMA (Table 1). The range of values reported corresponds to modeling with the highest dose included (0.42 W/kg for CDMA and 0.29 W/kg for GSM) and excluded (0.27 W/kg for CDMA and 0.2 W/kg for GSM).

**Table 1:** BMD_10_ and BMDL_10_ SAR values calculated with Bayesian model averaging for all sites cardiomyopathy in male rats following 19 weeks of exposure

	**CDMA**	**GSM**
BMD_10_, all doses	1.75 W/kg	0.94 W/kg
BMDL_10_, all doses	0.42 W/kg	0.29 W/kg
BMD_10_, the highest dose omitted	0.97 W/kg	0.58 W/kg
BMDL_10_, the highest dose omitted	0.27 W/kg	0.20 W/kg

BMD modeling of right ventricle cardiomyopathy data for female and male rats following a 2-year exposure resulted in consistently lower BMD and BMDL values for males compared to females, indicating greater susceptibility of males to RFR-induced cardiomyopathy (Supplementary Tables 1 and 2). BMDL_10_ for right ventricle cardiomyopathy in females corresponded to 2.7–5.16 W/kg whole-body SAR for CDMA and 1.91–2.18 W/kg for GSM modulation (Table 2). In male rats, the corresponding BMDL_10_ values were 0.7–0.79 W/kg whole-body SAR for CDMA and 0.33–0.42 W/kg for GSM modulation (Table 2).

**Table 2:** BMD_10_ and BMDL_10_ SAR values calculated with Bayesian model averaging for right ventricle cardiomyopathy in male and female rats following a 2-year exposure

	**Females** **CDMA**	**Females** **GSM**	**Males** **CDMA**	**Males** **GSM**
BMD_10_, all doses	10.68 W/kg^a^	4.86 W/kg	1.50 W/kg	0.81 W/kg
BMDL_10_, all doses	5.16 W/kg	2.18 W/kg	0.70 W/kg	0.42 W/kg
BMD_10_, the highest dose omitted	5.21 W/kg	2.94 W/kg	1.69 W/kg	0.69 W/kg
BMDL_10_, the highest dose omitted	2.70 W/kg	1.91 W/kg	0.79 W/kg	0.33 W/kg

^a^ Calculated BMD_10_ value is greater than the 6 W/kg highest SAR exposure dose in the study

**Fig. 5:**
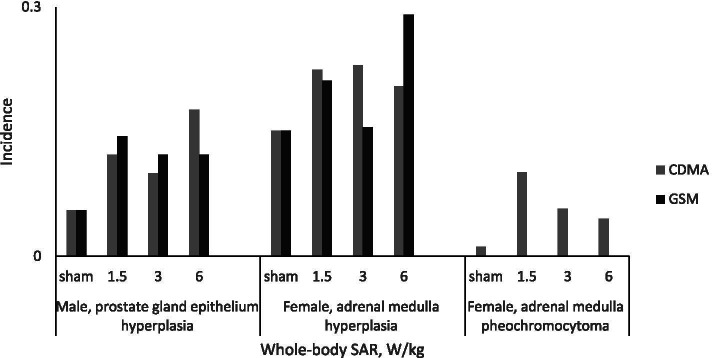
Prostate gland hyperplasia in male rats and adrenal medulla effects in female rats at 2 years

**Fig. 6:**
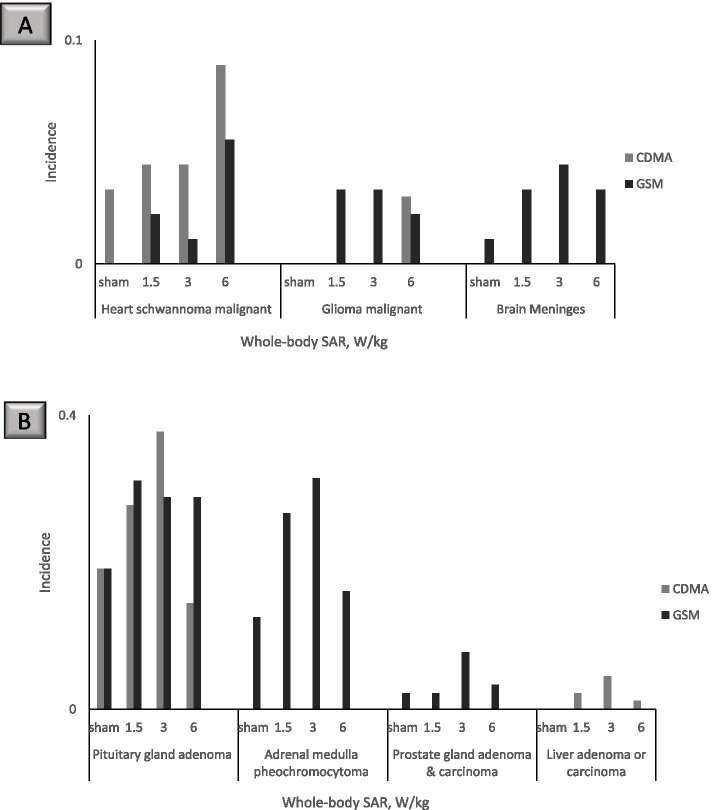
Neoplasm incidence in male rats at 2 years. Where no bar is shown, the specific outcome in question was not observed in the animal group. **A** Heart and brain tumors. **B** Pituitary gland, adrenal medulla, prostate gland, and liver tumors

Non-monotonic dose–response relationships were also observed in the NTP dataset for hyperplasias (Fig. 5). The incidence of prostate gland hyperplasia in male rats and adrenal medulla effects in female rats increased and decreased along the range of exposure doses tested (Fig. 5). A complex dose–response relationship also was seen for the tumors in the heart, brain, pituitary gland, adrenal medulla, prostate gland, and liver. For certain outcomes, animals in the 6 W/kg exposure group had the highest incidence, such as heart schwannomas in CDMA- and GSM- exposed male rats (Fig. 6A). In contrast, a decrease in incidence in the 6 W/kg groups was observed for several neoplasms, such as adrenal medulla neoplasms in CDMA-exposed female rats (Fig. 5), pituitary gland adenomas in CDMA-exposed male rats (Fig. 6B), and adrenal medulla and prostate gland neoplasms in GSM-exposed male rats (Fig. 6B).

Frequentist modeling of benchmark values for adrenal medulla hyperplasia in female rats and prostate gland epithelium hyperplasia in male rats following a 2-year exposure resulted in BMD_10_ higher than BMD_10_ values for cardiomyopathy (Supplementary Table 3). Most of the modeled BMDL_10_ values for these two health outcomes were in the range of 1–3 W/kg whole-body SAR. Similar to the greater sensitivity of males to RFR-induced cardiomyopathy compared to females, prostate gland epithelium hyperplasia in males was a more sensitive outcome than adrenal medulla hyperplasia in females (Fig. 5, Supplementary Table 3).

Following the approaches recommended by the U.S. EPA BMDS technical guidance [47] and the published methods for tumor data modeling from the California Environmental Protection Agency Office of Environmental Health Hazard Assessment [51], we modeled the benchmark values for specific neoplasms as well as multi-site tumor incidence in male rats. Neoplasm incidences in female rats could not be modeled. For male rats, the non-monotonic dose–response relationship patterns precluded BMDS modeling of certain tumor outcomes using all exposure dose groups (Supplementary Tables 4 and 5). Using all exposure dose groups, heart schwannoma data for male rats could be modeled for both CDMA and GSM exposures with 5% and 10% change in response rate above background (Table 3). With 10% response modeling for heart schwannoma, calculated BMD_10_ values exceeded the SAR value of 6 W/kg for the highest exposure dose group in the study, suggesting that the 10% response modeling is not practical for some tumor types in this dataset.

**Table 3:** BMDS modeling of cancer incidence in male rats using the MS Combo model. Neoplasm incidence data analyzed here included all exposure groups

Exposure condition and tumor type	BMD_5_	BMDL_5_	BMD_10_	BMDL_10_
CDMA, heart schwannoma	4.29	2.71	8.82^a^	5.40
CDMA, pituitary gland adenoma	Could not be modeled			
CDMA, liver adenoma	Could not be modeled			
**CDMA, combined heart schwannoma, liver adenoma, and pituitary gland adenoma**	**Could not be modeled**			
GSM, heart schwannoma	5.94	3.49	11.82^a^	6.20
GSM, brain malignant glioma	9.00^a^	3.60	Could not be modeled	
GSM, brain meninges neoplasm (benign and malignant)	11.31^a^	3.89	Could not be modeled	
GSM, adrenal medulla pheochromocytoma (benign and malignant)	Could not be modeled			
GSM, pituitary gland adenoma	2.86	1.15	Could not be modeled	
**GSM, combined heart schwannoma, brain glioma, and meninges neoplasm, adrenal pheochromocytoma, and pituitary gland adenoma**	**Could not be modeled**			

^a^ Calculated BMD value is greater than the 6 W/kg highest exposure dose in the NTP study

The omission the highest exposure dose group and the use of 5% BMR as the modeling approach allowed the modeling of more tumor outcomes, providing an acceptable fit to the remaining dose–response data (Supplementary Table 5 and Table 4). Omission of the highest exposure dose also allowed the modeling of combined incidences of different tumors, which is a modeling option provided in the U.S. EPA BMDS software (Table 4). Modeling of combined tumor outcomes allowed for greater sensitivity and resulted in lower BMD values compared to modeling of individual tumor incidences. Even with the omission of the highest dose, BMD_10_ values could not be modeled for heart schwannoma, brain meninges neoplasm, and combined tumors for GSM exposure (Table 4).

**Table 4:** BMDS modeling of cancer incidence in male rats using the MS Combo model. Neoplasm incidence data modeled here did not include the highest exposure dose of 6 W/kg

Exposure condition and tumor type	BMD_5_	BMDL_5_	BMD_10_	BMDL_10_
CDMA, heart schwannoma	4.09^a^	2.12	8.41^a^	3.50
CDMA, pituitary gland adenoma	0.60	0.37	1.23	0.77
CDMA, liver adenoma	3.37^a^	1.84	6.93^a^	3.78
**CDMA, combined heart schwannoma, liver adenoma, and pituitary gland adenoma**	**0.45**	**0.31**	**0.93**	**0.63**
GSM, heart schwannoma	6.87^a^	3.01	Could not be modeled	
GSM, brain malignant glioma	3.40^a^	1.86	6.99^a^	3.46
GSM, brain meninges neoplasm (benign and malignant)	4.21^a^	1.90	Could not be modeled	
GSM, adrenal medulla pheochromocytoma (benign and malignant)	0.58	0.38	1.20	0.78
GSM, pituitary gland adenoma	1.06	0.51	2.17	1.06
**GSM, combined heart schwannoma, brain glioma, and meninges neoplasm, adrenal pheochromocytoma, and pituitary gland adenoma**	**0.30**	**0.21**	**Could not be modeled**	

^a^ Calculated BMD value is greater than the 3 W/kg highest exposure dose included in this analysis

The combined neoplasm incidence data could be modeled with a 5% increased incidence above background in the dataset without the highest dose (Table 4). BMDL_5_ value of 0.31 W/kg whole-body SAR was calculated for combined tumors in CDMA-exposed rats and BMDL_5_ value of 0.21 W/kg was calculated for GSM. These BMDL_5_ values for combined tumor incidence are overall comparable to the BMDL_10_ values for cardiomyopathy effects. Of note, throughout our analysis, BMD and BMDL values were consistently lower for GSM-exposed animals relative to CDMA exposures, indicating greater sensitivity to GSM modulation in the NTP study.

## Discussion

Radiofrequency radiation can elicit carcinogenic, genotoxic, reproductive, developmental, neurological, and cognitive effects [52–54]. Continuously increasing exposure to radiofrequency radiation from wireless communication devices and sources brings urgency to the question of health-protective limits for such exposures. Here we use benchmark dose modeling as an approach to develop health-based exposure limits for RFR based on animal toxicology data. The lower limit on the modeled benchmark dose, abbreviated as BMDL, offers a 95% statistical estimate on the exposure dose associated with a 5% or a 10% change in response rate, which can be treated as a point of departure for the development of health-based exposure guidelines. To extrapolate from a point of departure from animal toxicological studies to the exposure level acceptable for the human population, safety factors are commonly used, including a ten-fold factor for differences between species (humans and laboratory animals, in this case) and a second tenfold factor for variability in the potential sensitivity within the human population [55]. The use of these two ten-fold safety factors comes from a decades-old practice in chemical risk assessment. It is possible that the range of variations in sensitivity within the human population and the range of differences between species may not be captured in the conventionally applied ten-fold factors. Further, to protect sensitive populations such as children, an additional safety factor may be necessary, an approach first introduced for pesticide risk assessment to account for susceptibilities during the vulnerable stages of early development [56].

**Fig. 7:**
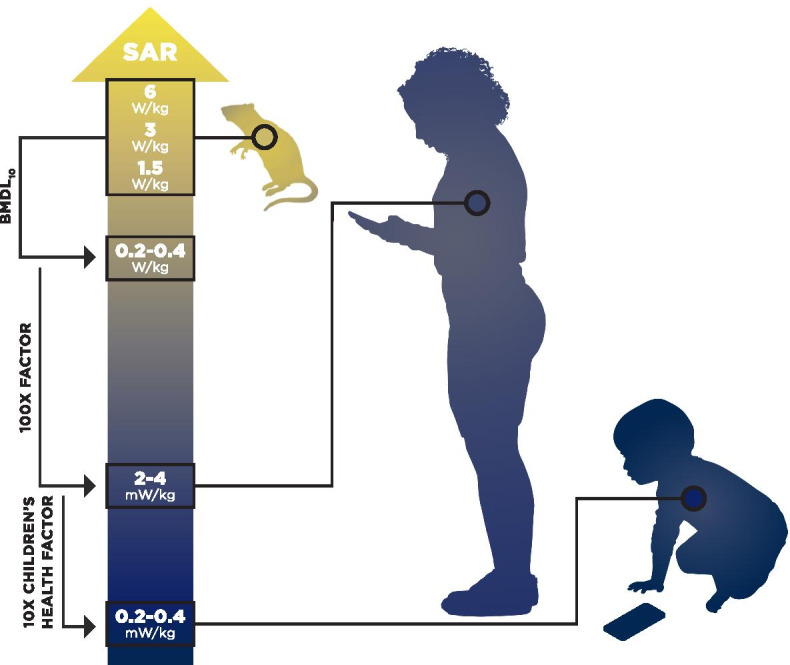
Summary of health-based exposure limits calculated in this study. Whole-body SAR value of 0.2–0.4 W/kg was selected as a point of departure for calculating health-based exposure guidelines. Applying two ten-fold safety factors for interspecies and intraspecies variability (a total of 100X), a whole-body SAR limit of 2–4 mW/kg is derived for adults. The application of an additional ten-fold (10X) children’s health factor suggests a limit of 0.2–0.4 mW/kg whole body SAR for young children

Among the BMDL values calculated in this study, the most sensitive values were BMDL_10_ for all sites cardiomyopathy at 19 weeks of exposure in male rats. The range of BMDL_10_ values 0.2 to 0.4 W/kg, listed in Table 1, offers a point of departure for defining exposure limits. The application of two ten-fold safety factors for interspecies and intraspecies variability (combined 100X factor) to the point of departure suggests a whole-body SAR limit of 2 to 4 mW/kg for adults (Fig. 7). These SAR values are 20- to 40-fold smaller than the current U.S. whole-body SAR limit of 0.08 W/kg. Application of an additional ten-fold safety factor to account for the greater sensitivity of the developing organism points to a whole-body SAR limit of 0.2 to 0.4 mW/kg for the young child.

We note two key differences between the health-based exposure limits presented here and the current U.S. regulations for radiofrequency radiation which set the limits of 0.08 W/kg for the whole-body SAR and the localized spatial peak of 1.6 W/kg SAR as averaged over 1 g of tissue. These differences are in the selection of the point of departure and the application of safety factors. The existing U.S. radiofrequency radiation standards were based on the 1986 recommendations of the U.S. National Council on Radiation Protection and Measurements and 1991 recommendations of the Institute of Electrical and Electronics Engineers [2, 40], which chose SAR value of 4 W/kg as the point of departure based on changes in animal behavior observed in studies conducted in the late 1970s and early 1980s.

The regulatory limit of 0.08 W/kg whole-body SAR was calculated by the application of two factors, a ten-fold factor and a five-fold factor (combined 50X) to the 4 W/kg SAR point of departure. While the first ten-fold factor accounted for the translation from animals to humans, the five-fold factor, proposed in the 1986 report by the U.S. National Council on Radiation Protection and Measurements, related to the difference in duration of work-related occupational exposure versus continuous exposure for the general public and not to health considerations [2]. The legal limit of 0.08 W/kg whole-body SAR did not include safety factors to account for potential variability in the sensitivity within the human population and greater sensitivity of children. The localized SAR limit of 1.6 W/kg was defined by multiplying the whole-body SAR limit of 0.08 W/kg by a factor of twenty [39, 40]. In the historical context of 1980s discussions of allowable exposure limits for occupational and general public exposures, the concept of a 20-fold increase of peak SAR relative to the whole-body SAR was based on the view that increase in tissue temperature was the main effect of radiofrequency radiation [40].

The epidemiological studies of mobile phone users and the reports of an elevated risk of head tumors on the side of the head where the phone was typically used highlight the toxicological importance of peak exposures in the part of the body closest to the wireless device [18–21, 57]. For example, as reported in a study from Israel, there was a 50–60% increased risk of parotid gland tumors on the ipsilateral side where hand-held mobile devices were used, while on the contralateral side, the risk of parotid gland tumors was not significantly different from controls [19]. Likewise, Hardell and coworkers reported a 90% increased risk of glioma for long-term ipsilateral exposure of mobile phones [57].

In the NTP study, laboratory animals received whole-body exposure to radiofrequency radiation from specially designed reverberation chambers [44, 45]. A dosimetry analysis for the animals in the NTP study reported the ratios between organ and whole-body SAR values [58]. According to that analysis, the peak SAR in the heart of male rats was 2.7 decibels (dB) higher than the whole-body SAR [58]. This difference of 2.7 dB between heart and whole-body SAR is much smaller than the 20-fold difference between localized and whole-body SAR assumed in the 1980s publications [39, 40].

In our view, more research is necessary to define what the acceptable, health-protective localized SAR value should be. An appropriate peak spatial SAR can be determined according to the concept of ALARA, or As Low As Reasonably Achievable, a concept developed for human exposures to ionizing radiation. Technical manuals for wireless devices specify that these products are tested for compliance with the SAR values defined under the U.S. Federal Communications Commission regulations, or under the corresponding regulations set by government authorities in other countries. However, investigative reports found that wireless devices may not comply with these regulatory limits if tested in a real-life scenario where the device touches the body directly [59].

Finally, the methodology used for the development of health-based exposure limits, such as the benchmark dose modeling and the application of safety factors, remains an evolving area of research in risk assessment. The lack of information about the shape of the dose–response curve below the lowest dose tested and the decreased incidence of various lesions observed at the highest dose tested by the NTP [42, 60, 61] pose questions for future research. The point of departure selected from benchmark modeling presented here, BMDL_10_ value of 0.2–0.4 W/kg whole-body SAR, does not correspond to the true “No Adverse Effects Level”, which is likely lower. Falcioni and coworkers reported a No Observed Adverse Effects Level of 0.03 W/kg for heart schwannomas in their study [7]. This NOAEL value is between the BMDL_10_ values calculated here and the health-based limit of 2–4 mW/kg for adults that we defined by the application of two ten-fold safety factors. While the use of ten-fold safety factors is an established risk assessment practice in the U.S. and other countries, it is possible that these standard factors either underestimate or overestimate the extent of variability in sensitivity within the human population. Even with the outstanding questions, the NTP and the Ramazzini Institute data on the RFR effects in laboratory animals are a valuable resource for the development of health-based guidelines for RFR exposures in people [41, 42].

## Conclusions

The analysis presented here supports a whole-body SAR limit of 2 to 4 mW/kg for adults, an exposure level that is 20- to 40-fold lower than the legally permissible limit of 0.08 W/kg for whole-body SAR under the current U.S. regulations. A ten-fold lower level of 0.2–0.4 mW/kg whole-body SAR may be appropriate for young children. Both technology changes and behavior changes may be necessary to achieve these lower exposure levels. Simple actions, such as keeping the wireless devices farther away from the body, offer an immediate way to decrease RFR exposure for the user.

## Supplementary Information

**Additional file 1: Supplementary Table 1**. Frequentist modeling of cardiomyopathy in male and female rats. Only models with good visual fit were included. Where several models produced identical BMD values, p values, scaled residuals and Akaike Information Criterion, all models giving the same calculated values are listed. **Supplementary Table 2**. 10% BMR estimates for cardiomyopathy calculated with the Bayesian model averaging approach. Model Average values highlighted in bold at the bottom of each row are presented in Tables 1 and 2 of the Results section. **Supplementary Table 3**. Frequentist modeling of hyperplasias in male and female rats following 2 years of exposure. Only models with good visual fit were included. **Supplementary Table 4**. 10% BMR estimates for multistage MS Combo modeling of neoplasm incidence data in male rats at 2 years. Only the datasets that could be modeled are included in this table. **Supplementary Table 5**. 5% BMR estimates for multistage MS Combo modeling of neoplasm incidence data in male rats at 2 years. Only the datasets that could be modeled are included in this table.

## Data Availability

The datasets analyzed for this study can be found in the published National Toxicology Program technical report https://ntp.niehs.nih.gov/ntp/htdocs/lt_rpts/tr595_508.pdf?utm_source=direct&utm_medium=prod&utm_campaign=ntpgolinks&utm_term=tr595 and on the website of the U.S. National Toxicology Program at https://ntp.niehs.nih.gov/whatwestudy/topics/cellphones/index.html.
