# Supplementary material for: Development of health-based exposure limits for radiofrequency radiation from wireless devices using a benchmark dose approach
Source: Environ Health. 2021 Jul 17;20:84. doi: 10.1186/s12940-021-00768-1 (PMC8286570; doi:10.1186/s12940-021-00768-1)
Supplement: Supplementary file 1 — Additional file 1: Supplementary Table 1. Frequentist modeling of cardiomyopathy in male and female rats. Only models with good visual fit were included. Where several models produced identical BMD values, p values, scaled residuals and Akaike Information Criterion, all models giving the same calculated values are listed. Supplementary Table 2. 10% BMR estimates for cardiomyopathy calculated with the Bayesian model averaging approach. Model Average values highlighted in bold at the bottom of each row are presented in Tables 1 and 2 of the Results section. Supplementary Table 3. Frequentist modeling of hyperplasias in male and female rats following 2 years of exposure. Only models with good visual fit were included. Supplementary Table 4. 10% BMR estimates for multistage MS Combo modeling of neoplasm incidence data in male rats at 2 years. Only the datasets that could be modeled are included in this table. Supplementary Table 5. 5% BMR estimates for multistage MS Combo modeling of neoplasm incidence data in male rats at 2 years. Only the datasets that could be modeled are included in this table. [file 12940_2021_768_MOESM1_ESM.docx]

Additional File 1

**Supplementary Table 1**. Frequentist modeling of cardiomyopathy in male and female rats. Only models with good visual fit were included. Where several models produced identical BMD values, p values, scaled residuals and Akaike Information Criterion, all models giving the same calculated values are listed.

| **Exposure conditions and health outcomes modeled** | - **Model(s)** | - **BMD_10_** - **W/kg** | - **BMDL_10_** - **W/kg** | **P**   - **values** | - **Scaled residual** | - **Akaike Information Criterion** |
| --- | --- | --- | --- | --- | --- | --- |
| - 19 weeks, male rats, CDMA, - all sites cardiomyopathy, all doses | - Log-logistic | 0.67 | 0.22 | 0.83 | -0.18 | 57.37 |
|  | - Gamma - Weibull - Multistage degree 1 - Multistage Degree 2 | - 0.98 | - 0.42 | - 0.75 | - 0.32 | - 57.58 |
| - 19 weeks, male rats, CDMA, - all sites cardiomyopathy, the highest dose omitted | - Logistic | - 0.72 | - 0.40 | - 0.78 | - -0.12 | - 43.62 |
|  | - Gamma - Weibull - Multistage degree 1 Multistage Degree 2 | - 0.54 | - 0.24 | - 0.88 | - -0.04 | - 43.56 |
| - 19 weeks, male rats, GSM, - all sites cardiomyopathy, all doses | - Gamma - Weibull - Multistage degree 1 Multistage Degree 2 | - 0.53 | - 0.28 | - 0.40 | - -0.26 | - 54.21 |
| - 19 weeks, male rats, GSM, - all sites cardiomyopathy, the highest dose omitted | - Logistic | - 0.45 | - 0.29 | - 0.74 | - 0.14 | - 40.20 |
| - 2 years, female rats, GSM, - right ventricle cardiomyopathy, all doses | - Log-logistic | - 3.83 | - 2.32 | - 0.55 | 0.77 | 255.31 |
|  | - Gamma - Weibull - Multistage degree 1 Multistage Degree 2 | - 3.98 | - 2.49 | - 0.51 | - 0.82 | - 255.48 |
| - 2 years, female rats, GSM, - right ventricle cardiomyopathy, the highest dose omitted | - Multistage degree 1 | - 2.57 | - 1.55 | - 0.96 | - 0.02 | - 173.05 |
| 2 years, male rats, GSM   - right ventricle cardiomyopathy, all doses | - Log-logistic | - 0.46 | - 0.26 | - 0.67 | - 0.02 | - 411.85 |
|  | - Weibull - Multistage degree 1 - Multistage Degree 2 | - 0.69 | - 0.46 | - 0.49 | - -0.29 | - 412.49 |
| - 2 years, male rats, GSM,   right ventricle cardiomyopathy, the highest dose omitted | - Logistic   Probit | - 0.53 - 0.55 | - 0.37 - 0.38 | - 0.72 - 0.75 | - 0.13 - 0.12 | - 326.94 - 326.91 |
| - 2 years, female rats, CDMA, - right ventricle cardiomyopathy, all doses | Log-logistic | 9.80 | 4.30 | 0.71 | -0.42 | 203.64 |
|  | - Gamma - Multistage degree 1 - Multistage Degree 2 - Weibull | - 9.72 | - 4.38 | - 0.70 | - -0.41 | - 203.67 |
| - 2 years, female rats, CDMA, right ventricle cardiomyopathy, the highest dose omitted | - Log-logistic | - 5.26 | - 2.38 | - 0.89 | - -0.07 | - 144.46 |
|  | - Gamma - Multistage Degree 1 - Multistage Degree 2 - Weibull | - 5.15 | - 2.40 | - 0.88 | - -0.07 | - 144.46 |
| - 2 years, male rats, CDMA, right ventricle cardiomyopathy, all doses | Multistage Degree 2 | - 2.00 | - 0.83 | - 0.15 | - -1.54 | - 4449.52 |
| - 2 years, male rats, CDMA, right ventricle cardiomyopathy, the highest dose omitted | - Multistage Degree 1 - Weibull - Logistic | - 1.55 - 2.80 - 1.49 | - 0.64 - 1.33 - 0.69 | - 0.02 - 0.18 - 0.02 | - -1.89 - 5.18 - -1.87 | - 366.85 - 363.33 - 366.74 |

**Supplementary Table 2**. 10% BMR estimates for cardiomyopathy calculated with the Bayesian model averaging approach. Model Average values highlighted in bold at the bottom of each row are presented in Tables 1 and 2 of the Results section.

| - **Exposure conditions and health outcomes modeled** | - **Models** | - **BMD_10_** - **W/kg** | - **BMDL_10_** - **W/kg** | - **Scaled residual** | - **Visual fit** |
| --- | --- | --- | --- | --- | --- |
| - 19 weeks, male rats, CDMA, all sites cardiomyopathy, all doses | - Dichotomous Hill - Gamma - Logistic - Log-Logistic - Log-Probit - Multistage degree 3 - Multistage degree 2 - Multistage Degree 1 - Probit - Quantal Linear - Weibull - **Model Average** | - 1.22 - 2.53 - 2.26 - 1.21 - 2.53 - 0.94 - 0.90 - 0.88 - 1.68 - 1.03 - 2.39 - **1.75** | - 0.06 - 0.46 - 0.88 - 0.15 - 0.49 - 0.53 - 0.52 - 0.49 - 0.82 - 0.47 - 0.52 - **0.42** | - 0.34 - 0.61 - 0.56 - 0.35 - 0.63 - 0.42 - 0.37 - 0.29 - 0.25 - 0.29 - 0.62 | - Fair - Poor - Poor - Good - Poor - Good - Good - Good - Fair - Good - Poor |
| - 19 weeks, male rats, CDMA, all sites cardiomyopathy, the highest dose omitted | - Dichotomous Hill - Gamma - Logistic - Log-Logistic - Log-Probit - Multistage degree 2 - Multistage Degree 1 - Probit - Quantal Linear - Weibull - **Model Average** | - 0.89 - 1.24 - 1.20 - 0.77 - 1.31 - 0.48 - 0.46 - 0.89 - 0.56 - 1.21 - **0.97** | - 0.06 - 0.28 - 0.48 - 0.12 - 0.38 - 0.28 - 0.26 - 0.44 - 0.26 - 0.33 - **0.27** | - 0.25 - 0.27 - 0.19 - 0.15 - 0.42 - 0.14 - 0.01 - 0.21 - -0.13 - 0.33 | - Good - Fair - Poor - Good - Fair - Good - Good - Poor - Good - Fair |
| - 19 weeks, male rats, GSM, all sites cardiomyopathy, all doses | - Dichotomous Hill - Gamma - Logistic - Log-Logistic - Log-Probit - Multistage degree 3 - Multistage degree 2 - Multistage Degree 1 - Probit - Quantal Linear - Weibull - **Model Average** | - 0.52 - 1.08 - 1.29 - 0.69 - 1.19 - 0.78 - 0.74 - 0.69 - 1.06 - 0.64 - 1.50 - **0.94** | - 0.04 - 0.21 - 0.61 - 0.07 - 0.23 - 0.43 - 0.41 - 0.38 - 0.60 - 0.32 - 0.25 - **0.29** | - -0.16 - -0.06 - -0.21 - -0.47 - 0.13 - 0.05 - -0.50 - -0.58 - -0.13 - -0.51 - -0.02 | - Fair - Poor - Poor - Fair - Fair - Good - Good - Good - Poor - Good - Poor |
| - 19 weeks, male rats, GSM, all sites cardiomyopathy, the highest dose omitted | - Dichotomous Hill - Gamma - Logistic - Log-Logistic - Log-Probit - Multistage degree 2 - Multistage Degree 1 - Probit - Quantal Linear - Weibull - **Model Average** | - 0.58 - 0.60 - 0.60 - 0.64 - 1.01 - 0.40 - 0.36 - 0.52 - 0.35 - 0.85 - **0.58** | - 0.06 - 0.16 - 0.32 - 0.09 - 0.31 - 0.22 - 0.20 - 0.31 - 0.18 - 0.23 - **0.20** | - -0.08 - -0.31 - -0.53 - -0.18 - 0.01 - -0.01 - -0.13 - -0.28 - -0.10 - -0.02 | - Fair - Good - Good - Fair - Fair - Good - Fair - Good - Fair - Fair |
| - 2 years, female rats, CDMA, right ventricle cardiomyopathy, all doses | - Dichotomous Hill - Gamma - Logistic - Log-Logistic - Log-Probit - Multistage degree 3 - Multistage degree 2 - Multistage Degree 1 - Probit - Quantal Linear - Weibull - **Model Average** | 10.73   - 9.13 - 11.95 - 5.97 - 7.43 - 3.61 - 3.72 - 4.05 - 9.09 - 6.47 - 6.79 - **10.68** | - 4.71 - 4.98 - 5.82 - 3.65 - 5.07 - 2.80 - 2.79 - 2.86 - 5.21 - 3.82 - 4.27 - **5.16** | - -0.31 - -0.43 - -0.14 - -1.22 - -0.67 - -0.35 - -0.35 - -0.27 - -0.40 - -1.01 - -0.98 | - Fair - Poor - Good - Good - Poor - Fair - Good - Good - Poor - Good - Fair |
| - 2 years, female rats, CDMA, right ventricle cardiomyopathy, the highest dose omitted | - Dichotomous Hill - Gamma - Logistic - Log-Logistic - Log-Probit - Multistage degree 2 - Multistage Degree 1 - Probit - Quantal Linear - Weibull - **Model Average** | - 5.00 - 4.40 - 6.04 - 3.07 - 3.53 - 1.96 - 2.16 - 4.52 - 3.50 - 3.38 - **5.21** | - 2.58 - 2.59 - 2.96 - 2.08 - 2.54 - 1.47 - 1.52 - 2.66 - 2.07 - 2.31 - **2.70** | - -0.06 - -0.19 - 0.13 - -0.98 - -0.54 - -0.80 - -0.74 - -0.11 - -0.70 - -0.74 | - Fair - Fair - Fair - Fair - Poor - Good - Good - Poor - Good - Fair |
| - 2 years, female rats, GSM, right ventricle cardiomyopathy, all doses | - Dichotomous Hill - Gamma - Logistic - Log-Logistic - Log-Probit - Multistage degree 3 - Multistage degree 2 - Multistage Degree 1 - Probit - Quantal Linear - Weibull - **Model Average** | - 3.86 - 4.68 - 5.86 - 3.54 - 4.67 - 2.85 - 2.80 - 2.83 - 5.17 - 3.69 - 4.21 - **4.86** | - 1.45 - 2.73 - 3.94 - 1.97 - 2.56 - 2.18 - 2.12 - 2.07 - 3.64 - 2.43 - 2.43 - **2.18** | - 0.67 - -0.67 - -0.21 - 0.60 - -0.93 - 0.29 - 0.25 - 0.27 - -0.44 - 0.64 - 0.86 | - Good - Fair - Good - Good - Fair - Fair - Good - Good - Poor - Good - Good |
| - 2 years, female rats, GSM, right ventricle cardiomyopathy, the highest dose omitted | - Dichotomous Hill Gamma - Logistic - Log-Logistic - Log-Probit - Multistage degree 2 - Multistage Degree 1 - Probit - Quantal Linear - Weibull - **Model Average** | - 2.76 - 2.78 - 3.24 - 2.31 - 2.72 - 1.61 - 1.67 - 2.84 - 2.29 - 2.53 - **2.94** | - 1.57 - 1.81 - 2.17 - 1.49 - 1.88 - 1.22 - 1.20 - 2.02 - 1.48 - 1.71 - **1.91** | - -0.04 - -0.11 - 0.23 - -0.65 - -0.49 - -0.71 - -0.70 - 0.02 - -0.33 - -0.44 | - Good - Fair - Good - Fair - Fair - Good - Good - Poor - Good - Fair |
| - 2 years, male rats, CDMA, right ventricle cardiomyopathy, all doses | - Dichotomous Hill - Gamma - Logistic - Log-Logistic - Log-Probit - Multistage degree 3 - Multistage degree 2 - Multistage Degree 1 - Probit - Quantal Linear - Weibull - **Model Average** | 1.77   - 1.44 - 0.95 - 1.79 - 2.14 - 1.11 - 1.00 - 0.79 - 0.94 - 1.80 - 1.91   **1.50** | - 0.92 - 0.73 - 0.70 - 0.91 - 1.24 - 0.73 - 0.66 - 0.56 - 0.71 - 0.56 - 0.96 - **0.70** | - -1.52 - -1.80 - -2.10 - -1.56 - -1.36 - -1.78 - -1.91 - -2.22 - -2.06 - -2.22 - -1.61 | - Fair - Fair - Good - Fair - Fair - Good - Good - Good - Good - Good - Fair |
| - 2 years, male rats, CDMA, right ventricle cardiomyopathy, the highest dose omitted | - Dichotomous Hill - Gamma - Logistic - Log-Logistic - Log-Probit - Multistage degree 2 - Multistage Degree 1 - Probit - Quantal Linear - Weibull - **Model Average** | - 1.84 - 1.85 - 1.43 - 1.62 - 2.01 - 0.81 - 0.78 - 1.25 - 1.11 - 1.75 - **1.69** | - 0.84 - 0.86 - 0.73 - 0.80 - 1.27 - 0.55 - 0.51 - 0.69 - 0.59 - 0.97 - **0.79** | - -1.56 - -1.71 - -1.87 - -1.43 - -1.12 - -1.95 - -2.06 - -1.87 - -1.94 - -1.46 | - Fair - Fair - Good - Poor - Poor - Good - Good - Good - Good - Fair |
| - 2 years, male rats, GSM, right ventricle cardiomyopathy, all doses | - Dichotomous Hill - Gamma - Logistic - Log-Logistic - Log-Probit - Multistage degree 3 - Multistage degree 2 - Multistage Degree 1 - Probit - Quantal Linear - Weibull - **Model Average** | 0.65   - 0.87 - 0.89 - 0.68 - 1.06 - 0.91 - 0.83 - 0.72 - 0.89 - 0.71 - 1.02 - **0.81** | - 0.13 - 0.26 - 0.62 - 0.13 - 0.31 - 0.59 - 0.54 - 0.49 - 0.65 - 0.48 - 0.25 - **0.42** | - -0.02 - -0.04 - -0.08 - -0.19 - 0.14 - 0.15 - 0.05 - -0.36 - -0.05 - -0.34 - 0.02 | - Fair - Good - Good - Good - Fair - Good - Good - Good - Good - Good - Good |
| - 2 years, male rats, GSM, - right ventricle cardiomyopathy, the highest dose omitted | - Dichotomous Hill - Gamma - Logistic - Log-Logistic - Log-Probit - Multistage degree 2 - Multistage Degree 1 - Probit - Quantal Linear - Weibull - **Model Average** | - 0.77 - 0.74 - 0.58 - 0.79 - 1.16 - 0.52 - 0.45 - 0.56 - 0.48 - 0.98 - **0.69** | - 0.16 - 0.28 - 0.39 - 0.20 - 0.46 - 0.34 - 0.31 - 0.39 - 0.31 - 0.34 - **0.33** | - -0.16 - -0.08 - -0.05 - -0.11 - 0.27 - 0.39 - 0.25 - 0.05 - 0.15 - 0.12 | - Fair - Fair - Good - Fair - Fair - Good - Good - Good - Good - Fair |

**Supplementary Table 3**. Frequentist modeling of hyperplasias in male and female rats following 2 years of exposure. Only models with good visual fit were included.

| - **Exposure conditions and health outcomes modeled** | - **Model(s)** | - **BMD_10_** - **W/kg** | - **BMDL_10_** - **W/kg** | **P**   - **values** | - **Scaled residual** | - **Akaike Information Criterion** |
| --- | --- | --- | --- | --- | --- | --- |
| - Female rats, CDMA, adrenal medulla hyperplasia, all doses | - Logistic - Weibull | - 46.27 - 45.76 | - 6.33 - 6.72 | - 0.03 - 0.03 | - -0.64 - -0.63 | - 151.56 - 151.56 |
| - Female rats, CDMA, adrenal medulla hyperplasia, the highest dose omitted | - Log-logistic | - 3.95 | - 3.02 | - 0.03 | - -1.19 | - 115.83 |
|  | - Gamma - Weibull - Multistage degree 1 - Multistage degree 2 | - 3.98 | - 2.17 | - 0.03 | - -1.17 | - 115.94 |
| - Female rats, GSM, adrenal medulla hyperplasia, all doses | - Logistic - Multistage degree 1 | - 4.43 - 4.32 | - 2.91 - 2.32 | - 0.33 - 0.29 | - -1.18 - -1.27 | - 353.61 - 353.84 |
| - Female rats, GSM, adrenal medulla hyperplasia, the highest dose omitted | - Logistic | - 61.24 | - 2.72 | - 0.24 | - -0.48 | - 248.9 |
| - Male rats, CDMA, prostate gland epithelium hyperplasia, all doses | - Log-logistic | - 5.02 | - 2.76 | - 0.51 | - 0.07 | - 248.52 |
|  | - Gamma - Weibull - Multistage degree 1 - Multistage degree 2 | - 5.07 | - 2.89 | - 0.50 | - 0.05 | - 248.54 |
| - Male rats, CDMA, prostate gland epithelium hyperplasia, the highest dose omitted | - Log-logistic | - 5.46 | - 2.17 | - 0.24 | - -0.56 | - 169.29 |
|  | - Gamma - Weibull - Multistage degree 1 - Multistage degree 2 | - 5.43 | - 2.22 | - 0.24 | - -0.55 | - 169.31 |
| - Male rats, GSM, prostate gland epithelium hyperplasia, all doses | - Log-logistic - Weibull | - 9.93 - 10.03 | - 3.72 - 3.91 | - 0.19 - 0.19 | - -0.56 - -0.54 | - 253.88 - 253.92 |
|  | - Gamma - Multistage degree 1 - Multistage degree 2 | - 10.04 | - 3.91 | - 0.19 | - -0.54 | - 253.92 |
| - Male rats, GSM, prostate gland epithelium hyperplasia, the highest dose omitted | - Log-logistic | - 3.48 | - 1.68 | - 0.18 | - -0.68 | - 185.53 |
|  | - Gamma - Weibull - Multistage degree 1 - Multistage degree 2 | - 3.51 | - 1.75 | - 0.17 | - -0.67 | - 185.60 |

**Supplementary Table 4**. 10% BMR estimates for multistage MS Combo modeling of neoplasm incidence data in male rats at 2 years. Only the datasets that could be modeled are included in this table.

| - **Exposure conditions and health outcomes modeled** | - **Model** | - **BMD_10_** - **W/kg** | - **BMDL_10_** - **W/kg** | **P**   - **values** | - **Scaled residual** | - **Akaike Information Criterion** |
| --- | --- | --- | --- | --- | --- | --- |
| - CDMA, heart schwannomas, all doses | - Multistage degree 2 | - 8.82 | - 5.40 | - 0.99 | - -0.09 | - 91.69 |
| - CDMA, heart schwannomas, the highest dose omitted | - Multistage degree 2 | - 8.41 | - 3.50 | - 0.95 | - -0.18 | - 47.58 |
| - CDMA, pituitary gland adenoma, the highest dose omitted^1^ | - Multistage degree 1 | - 1.23 | - 0.77 | - 0.82 | - -0.18 | - 316.54 |
| - CDMA, liver adenoma, the highest dose omitted^1^ | - Multistage degree 1 | - 6.93 | - 3.78 | - 0.98 | - 0.02 | - 55.82 |
| - GSM, heart schwannomas, all doses^2^ | - Multistage degree 1 | - 11.82 | - 6.20 | - 0.23 | - 0.21 | - 76.30 |
| - GSM, brain malignant glioma, the highest dose omitted^1^ | - Multistage degree 2 | - 6.99 | - 3.46 | - 0.69 | - -0.50 | - 55.32 |
| - GSM, adrenal medulla benign and malignant pheochromocytoma, the highest dose omitted^1^ | - Multistage degree 1 | - 1.20 | - 0.78 | - 0.46 | - 0.60 | - 286.08 |
| - GSM, pituitary gland adenoma, the highest dose omitted^1^ | - Multistage degree 1 | - 2.17 | - 1.06 | - 0.22 | - 0.99 | - 312.08 |

1. These datasets could only be modeled with the highest dose omitted.

2. Heart schwannoma dataset in GSM-exposed male rats could not be modeled with the highest dose omitted.

**Supplementary Table 5**. 5% BMR estimates for multistage MS Combo modeling of neoplasm incidence data in male rats at 2 years. Only the datasets that could be modeled are included in this table.

| - **Exposure conditions and health outcomes modeled** | - **Model** | - **BMD_5_** - **W/kg** | - **BMDL_5_** - **W/kg** | **P**   - **values** | - **Scaled residual** | - **Akaike Information Criterion** |
| --- | --- | --- | --- | --- | --- | --- |
| CDMA, heart schwannomas, all doses | - Multistage degree 1 | 4.29 | - 2.71 | - 0.99 | - -0.10 | - 91.69 |
| - CDMA, heart schwannomas, the highest dose omitted | Multistage degree 2 | - 4.09 | - 2.12 | - 0.95 | -0.18 | - 47.58 |
| - CDMA, pituitary gland adenoma, the highest dose omitted^1^ | - Multistage degree 1 | - 0.60 | - 0.37 | - 0.83 | - 0.07 | - 316.54 |
| - CDMA, liver adenoma, the highest dose omitted^1^ | - Multistage degree 1 | - 3.37 | - 1.84 | - 0.98 | - 0.02 | - 55.82 |
| - GSM, heart schwannomas, all doses | - Multistage degree 1 | - 5.94 | - 3.49 | - 0.70 | - 0.22 | - 72.30 |
| - GSM, heart schwannomas, the highest dose omitted | - Multistage degree 2 | - 6.87 | - 3.01 | - 0.47 | - 0.71 | - 33.57 |
| - GSM, brain malignant glioma, all doses | - Multistage degree 1 | - 9.00 | - 3.60 | - 0.17 | - 0.89 | - 79.70 |
| - GSM, brain malignant glioma, the highest dose omitted | - Multistage degree 2 | - 3.40 | - 1.86 | - 0.69 | - -0.50 | - 55.32 |
| - GSM, brain meninges neoplasm (benign and malignant), all doses | - Multistage degree 1 | - 11.31 | - 3.89 | - 0.54 | - -0.55 | - 101.59 |
| - GSM, brain meninges neoplasm (benign and malignant), the highest dose omitted | - Multistage degree 1 | - 4.21 | - 1.90 | - 0.81 | - -0.13 | - 74.08 |
| - GSM, adrenal medulla pheochromocytoma (benign and malignant), the highest dose omitted^1^ | - Multistage degree 2 | - 0.58 | - 0.38 | - 0.46 | - -0.21 | - 286.08 |
| - GSM, pituitary gland adenoma, all doses | Multistage degree 1 | 2.86 | 1.15 | 0.25 | 0.28 | 421.57 |
| - GSM, pituitary gland adenoma, the highest dose omitted | - Multistage degree 1 | - 1.06 | - 0.51 | - 0.22 | - 0.99 | - 312.08 |

1. These datasets could only be modeled with the highest dose omitted.
